# Serum adiponectin-levels are predictive of probable posttraumatic stress disorder in women

**DOI:** 10.1016/j.ynstr.2022.100477

**Published:** 2022-08-08

**Authors:** Eileen Vuong, Shibe Mhlongo, Esnat Chirwa, Carl Lombard, Nasheeta Peer, Sian Megan Hemmings, Naeemah Abrahams, Soraya Seedat

**Affiliations:** a South African Research Chairs Initiative (SARChI), PTSD Program, Department of Psychiatry, Stellenbosch University, South Africa; b Department of Psychiatry, Stellenbosch University, Stellenbosch, South Africa; c Gender and Health Research Unit, South African Medical Research Council, Tygerberg, South Africa; d Biostatitistics Unit, South African Medical Research Council, South Africa; e Non-Communicable Diseases Research Unit, South African Medical Research Council, and Department of Medicine, University of Cape Town, South Africa; f South African Medical Research Council / Stellenbosch University Genomics of Brain Disorders Research Unit, Stellenbosch University, Cape Town, South Africa; g School of Public Health and Family Medicine: Faculty of Health Sciences, University of Cape Town, South Africa; h School of Public Health, Faculty of Health Sciences, University of Witwatersrand, South Africa

**Keywords:** Adiponectin, Biomarker, Post-traumatic stress disorder, Posttraumatic stress symptoms, Rape, Trauma

## Abstract

**Background:**

Accumulative evidence indicates a role for adiponectin, a polypeptide secreted by adipose tissue, in the pathophysiology of posttraumatic disorder (PTSD) via metabolic and inflammatory pathways. This study examined adiponectin as a potential predictive biomarker for PTSD among female rape survivors.

**Methods:**

We evaluated the relationship of baseline serum adiponectin levels to the development of probable PTSD at 3- and 6-months post rape-exposure and compared adiponectin levels between 542 rape-exposed (RE) and 593 rape-unexposed women (RUE). Probable PTSD were defined as Davidson Trauma Scale score ≥40. Data were analysed using multivariate regression models and a generalized estimating equation (GEE) model. We adjusted for clinically relevant covariates associated with PTSD, as well as adiposity indices.

**Results:**

Participants who were in the mid-and high adiponectin tertile groups versus the lowest tertile group had a significantly reduced risk of probable PTSD among at 6 months follow-up, independent of adiposity(aOR = 0.45[0.22–1.05], p = 0.035; aOR = 0.44[0.22–0.90], p = 0.024). However, there was no effect of group (RE vs. RUE).

**Limitations:**

Adiponectin assays were conducted on non-fasting blood samples and information on chronic medication, dietary factors and levels of physical activity were not collected. There was a high attrition rate among rape exposed participants.

**Conclusions:**

Our results show that higher serum adiponectin levels are associated with reduced risk of probable PTSD over a 6-month period. This finding supports the hypothesis that serum adiponectin is a potential risk biomarker for PTSD.

## Highlights

• The prospective associations between adiponectin, an anti-inflammatory cytokine and development of PTSD was examined in a cohort of rape exposed women.
• Serum adiponectin levels were inversely associated with probable PTSD at 6 month among control but not rape exposed women.
• Results suggest that adiponectin may serve as a potential risk biomarker of PTSD.

## 1 Introduction

Rape exposure is a common traumatic experience, globally and in South Africa (Dartnall and Jewkes, 2013; Machisa et al., 2011). Compared to other traumatic events, rape and other sexual assault are associated with the greatest risk of posttraumatic stress disorder (Dworkin, 2018). Whilst estimates vary, approximately half of rape victims develop PTSD (Breslau, 2009; Kessler et al., 1995). PTSD can only be diagnosed after four weeks of persistent symptoms across four symptom clusters: intrusion, avoidance, negative alterations in cognition and mood, and alterations in arousal and reactivity, following the index trauma (American Psychiatric Association, 2013). As early intervention can prevent symptom chronicity, there is a need to better identify those individuals who are at the highest risk for subsequent PTSD (Oosterbaan et al., 2019). The defined temporal relationship between trauma exposure and the development of PTSD affords a unique opportunity to examine risk biomarkers for disease onset.

Although no valid and reliable PTSD biomarkers have been identified to date (Michopoulos et al., 2016), a strong body of evidence supports a role for metabolic and inflammatory mechanisms in PTSD pathophysiological processes (Speer et al., 2018). Decreased activity of the hypothalamic-pituitary-adrenal (HPA) axis and parasympathetic nervous systems, along with increased activity of the sympathetic nervous system, have been observed in individuals with PTSD (de Kloet et al., 2005; Speer et al., 2018). These may lead to increased levels of proinflammatory cytokines, such as tumour necrosis factor-α, interferon γ, C-reactive protein, interleukin-1β and interleukin-6 (Cavalcante Passos et al., 2015). Adiponectin is a metabolically active fat-derived cytokine with anti-inflammatory, anti-atherogenic and insulin-sensitizing properties (Ohashi et al., 2012). Adiponectin-regulation and signalling have been widely studied in multisystem inflammatory illnesses and shown to be negatively associated with obesity and diabetes, both well-established comorbidities in PTSD (Farr et al., 2014; Koenen et al., 2017; Rao et al., 2014). Interestingly, adiponectin also appears to be altered in several mental illnesses where metabolic and inflammatory aetiologies are dually implicated, including PTSD (Bloemer et al., 2018; Hryhorczuk et al., 2013; Vuong et al., 2020).

Despite interest in the associations between inflammation, metabolic disease and PTSD, few studies have investigated anti-inflammatory markers, such as adiponectin as risk predictors of PTSD. In a sample of 507 male firefighters, lower plasma adiponectin levels were found among participants with PTSD symptoms (PTSS) compared to controls (Na et al., 2017). Notably, no longitudinal study conducted in trauma-exposed individuals early after trauma have examined the associations of circulating adiponectin levels and mental health outcomes, and specifically none from low- and middle-income countries (LMIC).

The aim of the current study was to investigate whether lower levels of serum adiponectin (s-ADP), precede and predict the development of probable PTSD, following rape-exposure in a sample of female rape survivors.

## 2 Methods

### 2.1 Participants and setting

This study is nested within the Rape Impact Cohort Evaluation (RICE) study, a large prospective comparative cohort study evaluating the impact of rape on women's physical and mental health and their use of health services in South Africa over a 3-year post rape-period (Abrahams et al., 2017). A detailed description of the methods used in the parent study has been published (Abrahams et al., 2017). In brief, RE participants were recruited from five post-rape service centres in South Africa that provide comprehensive emergency care, including access to police, counselling, and medical and forensic care (Jordaan and Slaven, 2016).

Interested individuals were invited to the study site where study procedures were explained, informed consent obtained, and baseline interviews and assessments completed. Recruitment was restricted to female participants aged 16–40 years who laid a complaint of rape occurring within the previous 20 days at a Rape Centre. The South African Criminal Law (Sexual Offences and Related Matters) Amendment Act 32 of 2007, defines rape as any unlawful and intentional form of oral, anal, or vaginal penetration without consent, irrespective of gender. Women recruited into this study all reported vaginal penetration.

A control group of RUE participants were drawn from women attending Primary Health Care (PHC) clinics, mainly Family Planning and Child Wellness services. Women who reported previous (lifetime) exposure to sexual violence, other than the recent rape (within 20 days), were identified with three screening questions at the baseline interview. Participants who indicated one/more of the following: intimate partner rape and/or non-partner rape and/or first sexual intercourse that was forced/rape; were excluded from the unexposed cohort. Recruitment took place between October 2014 and June 2019. Participants in both the case and control groups were excluded if they were too emotionally distressed at the time of enrolment to provide informed consent, intellectually disabled or more than 14 weeks pregnant.

In total, the parent study enrolled 1799 female participants (N = 852 rape-exposed and N = 947 rape unexposed). At the baseline and follow up visits, RE and RUE participants completed the same set of mental health assessments, including self-report measures of risk and protective factors for the development of PTSD. In this study, all self-report measures were read out aloud to participants by trained research assistants supervised by a senior psychiatrist. Participants also underwent a physical examination and provided blood samples. Both cohorts were followed up every 3 months in the 1st year and every 6 months thereafter.

**Fig. 1:**
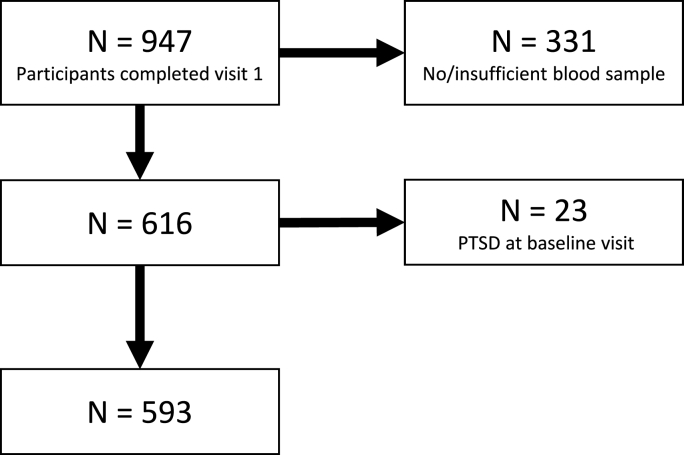
Participant selection (A) Rape unexposed control group participants, (B) Rape exposed participants.

For the current sub-study, we included all RE and RUE participants with blood samples for s-ADP assays. We excluded women who met criteria for PTSD on the Mini International Neuropsychiatric Interview (MINI) at the baseline assessment (i.e. presenting with PTSD due to a past traumatic event other than the rape), see Fig. 1. RE participants were recruited within 20 days of the rape incident, on average mean (sd): 11.88 (4.90) days.

### 2.2 Adiponectin assays

At baseline, non-fasting venous blood samples were drawn by a registered nurse. Samples were kept on ice and sent to the testing laboratory within 2–4 h. Whole blood fractions were separated by centrifuging the sample for 5 min at 3000 rpm at 4 °C, aliquoted into individual tubes and stored at −80 °C until the day of adiponectin level analysis using ELISA. Serum adiponectin levels were measured by using a commercially available human adiponectin enzyme-linked immunosorbent assay kit (Quantikine human adiponectin immunoassay, catalogue item DRP300 from R&D Systems Inc., Minneapolis, MN). All procedures were performed in strict accordance with the manufacturer's instructions. The assay conditions were controlled and standardised and the kits were from the same lot to optimize reproducibility. The average inter-assay and intra-assay coefficients of variations were 5.5% and 6.0% for adiponectin.

### 2.3 Clinical measures

#### 2.3.1 PTSD

##### 2.3.1.1 The Mini International Neuropsychiatric Interview (MINI)

The MINI version 7.0.0, a structured psychiatric interview that screens for 16 DSM-IV Axis I psychiatric disorders, was administered at the baseline visit to assess for a diagnosis of PTSD based on past trauma (i.e trauma prior to the index rape) (Sheehan et al., 2014). The MINI has shown good reliability and validity in various populations (de Azevedo Marques and Zuardi, 2008; Lecrubier et al., 1997).

##### 2.3.1.2 The Davidson Trauma Scale (DTS)

The Davidson Trauma Scale (DTS), a 17-item self-reporting and rating questionnaire that measures DSM-IV symptoms of PTSD within three specific clusters (intrusion, avoidance/numbing, and hyperarousal), was used to assess PTSD symptoms (PTSS) (Davidson et al., 1997) at all timepoints. RE participants completed the DTS in relation to the recent rape event. Responses were measured on a 5-point Likert scale for symptom frequency ranging from 0 (‘not at all’) to 4 (‘every day’) and symptom severity ranging from 0 (‘not at all distressing’) to 4 (‘extremely distressing’). An uploading error resulted in missing value sets for the symptom severity subscale of the DTS which was corrected once it was identified. There were no missing values for the DTS frequency subscale. In the present study, participants with both severity and frequency scores (i.e., those participants without missing data in the DTS scale) had either the same score for severity than for frequency, or very close to their frequency score. Missing DTS symptom severity values were imputed in the RICE study using a multiple imputation model while maintaining the multivariate normal distribution. The symptom frequency and severity scores were added together to produce a total PTSS score ranging between 0 and 136. A total score of 40 or more was considered indicative of probable PTSD (i.e., a PTSD symptom diagnosis). Symptom clusters were scored separately. The DTS has shown excellent discriminating power for distinguishing between participants with and without PTSD at a cut-point of 40 (Davidson et al., 1997). The DTS showed excellent reliability in this study at each timepoint with a Cronbach alpha sore of 0.92 at baseline, 0.91 at 3-months and 0.93 at 6-months post-rape.

#### 2.3.2 Other trauma-exposures

##### 2.3.2.1 Life Events Checklist (LEC)

A modified version of the LEC was used to measure lifetime exposure to different trauma types at the baseline visit (Jewkes et al., 2006; Weathers et al., 2013). The modified version of the LEC measures direct exposure to ten trauma types using a dichotomous ‘yes/no’ response. The trauma types measured were (1) imprisonment, (2) civil unrest/war, (3) serious injury, (4) being close to death, (5) murder of a family member or friend, (6) unnatural death of a family member or friend, (7) murder of a stranger/s, (8) robbed at gunpoint or knifepoint, (9) kidnapping and (10) sexual assault. An item from the modified version (“torture”) was not completed accurately by participants. Torture is not well translated in isiZulu and the meaning of “torture” was therefore misunderstood and misinterpreted. This item was therefore excluded from calculation of the total score on the LEC-modified version. The number of ‘yes’ responses from the remaining trauma types were added together to yield a total LEC score ranging from 0 to 10, indicating the cumulative lifetime trauma load of participants (Mollica et al., 1993). This continuous value was used in subsequent analysis, and no analysis of the different trauma-types was performed. The LEC has shown good convergence when compared to other established measures of trauma exposure (Gray et al., 2004). The Cronbach alpha score was 0.61 at baseline.

##### 2.3.2.2 Childhood Trauma Questionnaire Short Form (CTQ-SF)

Childhood trauma (before the age of 18 years) was measured using a modified version of the CTQ-SF (Bernstein et al., 1994; Chirwa et al., 2018). The modified version consists of 14 items and measures five type of childhood trauma, namely (i) witness of abuse of mother, (ii) sexual abuse, (iii) physical abuse, (iv) emotional abuse, and (v) parental neglect. Responses were measured on a 4-point Likert scale ranging from 1 (‘never’) to 4 (‘very often’). The modified CTQ-SF showed acceptable reliability in this study with a Cronbach alpha score of 0.75 at baseline.

#### 2.3.3 Other psychiatric disorders

##### 2.3.3.1 Center for Epidemiologic Studies Depression Scale (CES-D)

The Center for Epidemiologic Studies Depression Scale (CES-D) was used to measure depression in accordance with DSM-IV criteria at all time points (Radloff, 1977). The CES-D is a twenty-item, self-report measure with responses measured on a 4-point Likert scale ranging from 0 (‘rarely or none of the time’) to 3 (‘most or all of the time’), total score range between 0 and 60 A cut-off score of ≥16 has been recommended as indicative of caseness for depression with good sensitivity and specificity and high internal consistency (Lewinsohn et al., 1997). The CES-D showed excellent reliability for a Cronbach-alpha score of 0.92 at baseline.

#### 2.3.4 Behavioural risk factors

##### 2.3.4.1 Alcohol Use Disorders Identification Test-Concise (AUDIT-C)

The Alcohol Use Disorders Identification Test-Concise Scale (AUDIT-C) was used to measure hazardous alcohol use at all time points (Saunders et al., 1993). The original AUDIT consists of 10-items with responses measured on a 5-point Likert scale and response options specific to each individual item. A score of three or more on the AUDIT-C (the first three items of the AUDIT) is considered indicative of hazardous drinking in women (Bush et al., 1998). The AUDIT-C has shown good reliability and validity in various settings (Bohn et al., 1995). The AUDIT-C showed good reliability in this study with a Cronbach alpha score of 0.83.

##### 2.3.4.2 Smoking status

Current and past use of tobacco products were assessed through screening questions in the health questionnaire. Daily smoking was defined as smoking ≥1 cigarette per day. Occasional smoking was defined as smoking cigarettes, but not daily. Current smoking in this study included daily and occasional cigarette smokers.

#### 2.3.5 Post-rape risk factors

##### 2.3.5.1 Perceived Stress Scale (PSS)

The PSS is a 10-item self-report scale used to measure an individual's appraisal of stressful situations and investigates elements of anxiety related to personal control, predictability, and overburdening (Cohen et al., 1983). In the original PSS, responses are measured using a 5-point Likert scale with response options ranging from 0 ‘never’ to 4 ‘very often’. In the present study response options were modified to 1 = ‘never’ and 4 = ‘many times’ to be consistent with the response options contained in other measures that participants completed. High scores on the PSS indicate high perceived stress. The PSS showed good reliability with a Cronbach-alpha score of 0.85.

##### 2.3.5.2 Rape Stigma Scale (RSS)

A modified version of the HIV Stigma Scale (Kalichman et al., 2005) was used to measure negative personal perceptions of rape (e.g., self-blame and blame from others, embarrassment, shame, and estrangement) at each time-point. The RSS is a nine-item scale and responses are measured on a four-point Likert scale ranging from 1 ‘never’ to 4 ‘many times’. A high score indicates high rape stigma. The original HIV Stigma Scale showed acceptable internal consistency, test-retest reliability, and construct validity (Berger et al., 2001). The modified RSS scale showed good reliability in this study with a Cronbach alpha score of 0.85.

#### 2.3.6 Protective factors

##### 2.3.6.1 The Connor-Davidson Resilience Scale (CD-RISC)

Resilience as a measure of stress coping ability was assessed using the Connor Davidson Resilience Scale (CD-RISC), a 25 item self-report scale measuring resilience constructs e.g., personal competence, tolerance of negative affect (Connor and Davidson, 2003). The CD-RISC has shown good to excellent internal consistency in previous studies, with Cronbach alpha scores ranging between 0.81 and 0.94 (Connor and Davidson, 2003). The CD-RISC showed good reliability in this study with a Cronbach alpha score of 0.89.

##### 2.3.6.2 Multidimensional Scale of Perceived Social Support (MSPSS)

The MSPSS is a 12-item self-report scale used to determine an individual's perception of available social support obtained from family and friends (Zimet et al., 1988). Modified response options ranged from 1 ‘strongly disagree’ to 4 ‘strongly agree’. The scale has shown good reliability, factorial validity as well as construct validity (Zimet et al., 1988). The scale also showed good reliability in this study with Cronbach alpha score of 0.88.

#### 2.3.7 Clinical and anthropometric measurements

Anthropometric and blood pressure assessments were performed by trained fieldworkers. Weight was measured on a calibrated scale with participants in light clothing, without shoes, and recorded to nearest 0.5 kg. BMI was calculated using Quetelet's formula as weight (in kilograms) divided by height (in square metres) (Nishida et al., 2010). The BMI was classified into underweight (≤18 kg/m^2^) normal (18–24.9 kg/m^2^), overweight (25–29.9 kg/m^2^) and obese (≥30 kg/m^2^). Waist circumference (WC) was measured in centimetres using a non-elastic tape measure at the end of a normal expiration at the midpoint between the lower border of the lowest rib and the upper border of the hip bone, with the measuring tape parallel to the floor. WC was measured to the nearest 0.1 cm. Hip circumference (HC) was measured around the widest portion of the buttocks, with the measuring tape parallel to the floor. WHR was calculated as WC divided by HC. Blood pressure (BP) was measured using a digital BP monitor (Omron, M6 Comfort, Netherland) with participants seated in a resting position for at least 5 min before measurement. Three measurements were taken 3 min apart, and the average of the 2nd and 3rd readings were used in the analysis. The term “hypertension” was applied to include both participants with an existing diagnosis of hypertension; as well as participants with a systolic BP ≥ 140 mmHg or diastolic BP ≥ 90 mmHg. Participants were classified as having “diabetes” if; (i) reported an existing diagnosis of diabetes; and/or (ii) the presence of an elevated Hb1Ac (WHO, 2011)

#### 2.3.8 Ethical considerations

The study was introduced to potential participants as the “Women's Health and Wellbeing study” in order to protect them from being identified as someone who has been raped. All participants provided written informed consent. Ethical approval for RICE was obtained from the South African Medical Research Council Ethics Committee (SAMRC; EC019/2013). Approval to conduct the sub-study was obtained from the Health Research Ethics Committee at Stellenbosch University (HREC; N08/02/040).

### 2.4 Statistical analyses

#### 2.4.1 Descriptive & bivariate analysis

All data were processed using STATA 16. To test whether the data were normally distributed or not, the Shapiro-Wilk test was used. Participants’ baseline characteristics by exposure group were summarized using the mean ± SD for continuous variables while categorical variables were presented as frequencies with percentages. For comparison of two normally distributed means, the *t*-test was used. Where variables were not normally distributed, the comparison was conducted using the Wilcoxon rank sum test. The Pearson Chi-square was used to compare categorical variables. To assess associations between baseline characteristics and retention status, as defined by three categories, namely drop-out (baseline visit only); intermittent (either missed month 3 or month 6 follow-up) or completed all visits, Analysis of Variance (ANOVA) was used for continuous variables and Pearson chi-square test for categorical variables (Supplementary Tables 1 and S1). Differences in adiponectin concentrations by rape exposure and probable PTSD status were further evaluated at each time point with tests following the rules set above.

##### 2.4.1.1 Imputation process

Multiple Imputation was used to impute for the DTS frequency and severity scores, and other post-traumatic factors of interest. This method follows a Bayesian iterative Markov chain Monte Carlo (MCMC) procedure which assumes all the variables in the imputation model have a multivariate normal distribution and uses a uniform prior distribution. The imputation model included group status (RE vs. RUE) and baseline previous traumatic experiences as covariates. Prior to using multiple imputation, missing data patterns were examined. The DTS overall score was then derived using imputed DTS frequency and severity scores and bounded to follow the range of the original scale and then dichotomised with participants having a score of 40 or above assigned to the PTSD group (i.e. probable PTSD based on symptom status).

##### 2.4.1.2 Adiponectin and PTSD

The statistical methods in multivariate and longitudinal analyses were computed on the fitted multiple imputed datasets. PTSD was treated as a dichotomous variable, with a DTS score of ≥40 indicating probable PTSD. Adiponectin was analysed as a categorical (3-way/tertiles: 1st tertile: 2.23–10.56 μg/mL, 2nd tertile: 10.59–14.66 μg/mL, 3rd tertile: 14.68–32.12 μg/mL) instead of a continuous variable after assessing the linear effect of s-ADP which was not evident. We further analysed baseline factors associated with adiponectin tertiles using ANOVA for continuous baseline factors & Pearson chi-square for categorical factors.

##### 2.4.1.3 Multivariate analyses

The relationship between serum adiponectin tertile levels and probable PTSD at each time point was determined simultaneously using a multivariate logistic regression model, taking the lowest tertile as a reference. The main model included rape exposure, s-ADP, the interaction of rape x s-ADP and BMI (Cohen et al., 2011; Suliman et al., 2016). We then adjusted for a range of potential confounding factors which were a priori selected with reference as risk factors for PTSD. Variables, including age (Ditlevsen and Elklit, 2010; Obata et al., 2013), food security (Whittle et al., 2019), HIV (Neigh et al., 2016; Tong et al., 2003), childhood trauma (Halligan and Grossman, 2001; Lehto et al., 2012), lifetime trauma (Briere et al., 2016; Suliman et al., 2009), depression (Hu et al., 2015; Yehuda et al., 2012) and perceived stress (Catabay et al., 2019) were included in the model to obtain the adjusted OR, 95% CI & p-value. After the inclusion of all listed variables, we conducted a backward elimination procedure to simplify our model. Multiple testing was conducted using the Benjami Hochberg adjustment at 10% false discovery rate and the adjusted p-value was used to detect statistical significance.

##### 2.4.1.4 Longitudinal analyses

We used a generalized estimating equation (GEE) model to examine the longitudinal relationship between s-ADP and probable PTSD. We used an unstructured correlation structure matrix to evaluate the effects of the covariates such as time and group on PTSD outcome. The GEE model outputs are presented as odds ratios; adjusted odds ratios; 95% confidence intervals and corresponding p-values. Depression, previous trauma exposure and perceived stress were treated as time-varying variables.

##### 2.4.1.5 Sensitivity analyses

Sensitivity analysis was performed on the final GEE model to compare estimates from Multiple Imputation analysis and from non-imputed data (available case analysis). This was done to assess whether there would be significant differences in model estimates under the different missing data assumptions and thus impact on conclusions drawn from the results from the two approaches (Supplementary Tables 2 and S2).

##### 2.4.1.6 Post-hoc (bivariate) analysis

We further assessed whether s-ADP tertile levels could predict the risk of individual PTSD symptom clusters of DSM-IV according to the DTS scale; namely (i) intrusion, (ii) avoidance/numbing, and (iii) hyperarousal; at 3- and 6- months for both RE and RUE using the negative binomial model. The incidence rate ratio (IRR) and 95% CI are reported in the Supplementary Table 3.

## 3 Results

### 3.1 Participant characteristics

The participant selection procedure is presented in Fig. 1. This study reports on findings from 542 RE and 593 RUE participants. Baseline socio-demographic, behavioural, mental health and biochemical characteristics are presented in Table 1. RUE participants were more likely to have higher mean adiposity (BMI, WC, WHR) measures than the RE participants (p < 0.001). There was a higher prevalence of HIV (p < 0.003) and hypertension (p = 0.029) among the RE versus RUE group. A significantly higher number of RE participants had baseline adiponectin levels in the lowest s-ADP tertile as compared to the RUE participants (p < 0.001), (Table 1).

**Table 1:** Baseline characteristics of the study cohort (N = 1135).

Characteristics	All Participants N = 1135	Rape Exposed N = 542 (47.8%)	Rape Unexposed N = 593 (52.3%)	
		mean (sd) or n (%)	mean (sd) or n (%)	p-value
**Sociodemographic** **characteristics**				
Age (years)	25.2 (5.4)	24.7 (5.3)	25.7 (5.5)	**0.002**
Relationship status, n(%)*				
Single	192 (16.9%)	107 (19.7%)	85 (14.4%)	
Relationship, not cohabiting	848 (74.8%)	390 (72.0%)	458 (77.4%)	0.051
Married or cohabiting	94 (8.3%)	45 (8.3%)	49 (8.3%)	
Employed	232 (20.4%)	144 (26.6%)	88 (14.8%)	**<0.001**
Completed Secondary education	677 (59.7%)	314 (57.9%)	363 (61.2%)	0.260
Food secure	925 (81.5%)	441 (81.4%)	484 (81.6%)	0.912
**Behavioural risk factors**				
Smoking status				
Non-smoker	1002 (88.4%)	467 (86.2%)	535 (90.4%)	0.083
Yes, occasionally	65 (5.7%)	36 (6.6%)	29 (4.9%)	
Yes, daily^a^	67 (5.9%)	39 (7.2%)	28 (4.7%)	
Alcohol use (AUDIT –C)				
Audit-C score	1.8 (2.5)	2.0 (2.5)	1.7 (2.4)	**0.002**
Audit-C score: median (IQR)	1.0 (3.0)	1.0 (3.0)	0.0 (3.0)	**0.036**
Low	803 (70.8%)	366 (67.5%)	437 (73.7%)	**0.023**
High	332 (29.3%)	176 (32.5%)	156 (26.3%)	
**Chronic medical illness,** n (%)				
HIV positive	491 (43.3%)	259 (47.8%)	232 (39.1%)	**0.003**
Hypertension	115 (10.1%)	66 (12.2%)	49 (8.3%)	**0.029**
Diabetes	38 (3.4%)	20 (3.7%)	18 (3.0%)	0.540
**Adiposity measures**				
Body Mass Index (BMI)	27.2 (6.7)	26.1 (5.9)	28.2 (7.1)	**<0.001**
Body Mass Index (BMI) Categories				
Underweight: BMI <18.5	62 (5.5%)	32 (5.9%)	30 (5.1%)	**<0.001**
Normal weight: BMI 18.5–24.9	446 (39.3%)	248 (45.8%)	198 (33.4%)	
Overweight: BMI 25–29.9	259 (22.8%)	132 (24.4%)	127 (21.4%)	
Obese: BMI≥30	368 (32.4%)	130 (24.0%)	238 (40.1%)	
Waist Circumference	84.9 (14.3)	82.4 (12.4)	87.2 (15.5)	**<0.001**
Waist-to-Hip Ratio	0.8 (0.10)	0.8 (0.1)	0.8 (0.1)	**<0.001**
**Previous trauma exposure**				
Childhood trauma score (CTQ-SF)	16.0 (3.0)	16.2 (3.4)	15.8 (2.6)	0.329
CTQ-SF: median (IQR)	15.0 (3.0)	15.0 (3.0)	15.0 (3.0)	0.343
Lifetime trauma score (LEC)	1.5 (1.7)	1.9 (1.7)	1.2 (1.5)	**<0.001**
LEC: median (IQR)	1.0 (2.0)	1.0 (2.0)	1.0 (2.0)	**<0.001**
**Mental Health factors**				
Depression score (CES-D)*	22.9 (14.6)	33.0 (12.6)	13.6 (9.3)	**<0.001**
Posttraumatic stress score (DTS)*	37.2 (36.8)	69.3 (29.6)	14.5 (21.1)	**<0.001**
**Individual post-rape risk factors**				
Perceived stress score (PSS) **	22.3 (5.3)	22.9 (5.7)	21.7 (4.9)	**<0.001**
Rape stigma score (RSS) **	20.6 (7.1)	20.6 (7.1)	NA	
**Protective factors**				
Resilience score (CD-RISC) **	75.0 (6.2)	74.5 (6.1)	75.4 (6.2)	**0.023**
Social support score (MSPSS) **	35.0 (4.7)	35.0 (4.9)	35.0 (4.4)	0.843
**Adiponectin serum level**				
Adiponectin tertile categories				
serum levels: 2.23–10.56	380 (33.5%)	210 (38.8%)	170 (28.7%)	**<0.001**
serum levels: 10.59–14.67	377 (33.2%)	145 (26.8%)	232 (39.1%)	
serum levels: 14.68–32.12	378 (33.3%)	187 (34.5%)	191 (32.2%)	

Footnote: ¶ Wilcoxon rank sum test used to assess differences between groups. ¶¶ Median test used to assess the differences using medians between groups. Values in bold indicate p-value ≤ 0.05. Data are expressed as mean (SD) for continuous variables with normal distribution, median (interquartile range) for continuous variables with skewed distribution, and N, (percentage) for categorical variables. ^a^Daily smoking refers to use of ≥1 cigarette per day. *N = 1134: NRE = 59; **N = 1131; NRE = 590 & RE = 541; ***N = 491; RE = 259 & NRE = 232. Abbreviations: Childhood Trauma Questionnaire Short Form (CTQ-SF); Davidson Trauma Scale (DTS); Life Events Checklist (LEC); The Connor-Davidson Resilience Scale (CD-RISC); Multidimensional Scale of Perceived Social Support (MSPSS); Perceived Stress Scale (PSS); Rape stigma scale (RSS); Alcohol Use Disorders Identification Test- Concise (AUDIT-C); Center for Epidemiologic Studies Depression Scale (CES-D).

### 3.2 Mental health outcomes

Two hundred and forty-four (45.6%) and 177 (32.7%) participants in the RE group met study criteria for probable PTSD at the 3- and 6- month follow up visits, respectively. RE participants exhibited higher DTS, CES-D and AUDIT-C scores compared to RUE participants at the 3- and the 6- month follow up (Table 2). Of note, 79(13.4%) and 83(14.0%) participants in the RUE group also met criteria for probable PTSD at the 3- and 6-month study visits, respectively, that was consequential to exposure to other traumas other than rape. Among these, the most common traumas experienced according to the LEC were (i) robbed/highjacked at gun or knifepoint (31%); (ii) unnatural death of family or friend (19.9%) and (iii) torture (19.8%).

**Table 2:** Longitudinal mental health outcomes (N = 1135).

	3 months		6 months	
	Rape Exposed (N = 542)	Rape Unexposed (N = 593)	Rape Exposed (N = 542)	Rape Unexposed (N = 593)
	Mean (95% CI)/n(%)	Mean (95% CI)/n(%)	Mean (95% CI)/n(%)	Mean (95% CI)/n(%)
**PTSD, n(%)**	244(45.6%)	79(13.4%)	177(32.7%)	83(14.0%)
DTS: mean	39.2 (36.2–42.2)	16.1 (14.3–17.8)	30.7 (27.6–33.9)	16.4 (14.4–18.3)
**MDD, n(%)**	60(11.2%)	21(3.5%)	35(6.5%)	10(1.6%)
CES-D: mean	18.2 (17.1–19.3)	14.2 (13.3–15.0)	16.1 (14.7–17.4)	14.2 (13.2–15.1)
**Harmful drinking, n(%)**	149(27.4%)	133(22.4%)	151(27.8%)	110(18.6%)
AUDIT-C: mean	1.6 (1.4–1.9)	1.3 (1.1–1.5)	1.7 (1.4–1.9)	1.2 (1.0–1.4)

Footnote: ^a^Participants were assigned to a PTSD symptom group based on a DTS total score of ≥40. ^b^A cut-off score of ≥16 on the Center for Epidemiologic Studies Depression Scale (CES-D) was used at all time points to screen for presence of clinically significant depression, MDD, major depressive disorder. ^c^Harmful drinking was assessed via participant scores on the Alcohol Use Disorders Identification Test-Concise (AUDIT-C), with a cut-off of 3 indicating hazardous drinking.

### 3.3 Retention and drop-out

In the RUE group, most participants 479 (80.8%) completed all three study visits, whereas only 235 (43.4%) in the RE group completed all three study visits, 117 (21.6%) missed one follow-up visit and 190 (35.1%) missed both follow-up visits. For RE participants, when stratified by study retention status, no significant differences were observed in terms of PTSD-status across the three retention groups (see Supplementary Tables 1 and S1 for details).

### 3.4 Association of baseline sociodemographic and clinical characteristics with baseline serum adiponectin tertiles among all participants

Among the RE participants, significant associations were shown between baseline s-ADP levels and adiposity measures (BMI, WC, WHR), depression and resilience scores (p < 0.001; p = 0.023; p = 0.023). In the RUE group, only lifetime trauma was significantly associated with baseline s-ADP levels (p = 0.017) (Table 3).

**Table 3:** Baseline factors associated with baseline serum adiponectin among all participants.

	Rape Exposed (RE)				Rape Unexposed (RUE)			
	ADP levels 2.23–10.56	ADP levels 10.59–14.66	ADP levels 14.68–32.12		ADP levels 2.23–10.56	ADP levels 10.59–14.66	ADP levels 14.68–32.12	
	n = 210 (38.8%)	n = 145 (26.8%)	n = 187 (34.5%)		n = 170 (28.7%)	n = 232 (39.1%)	n = 191 (32.2%)	
	mean (sd) or n (%)	mean (sd) or n (%)	mean (sd) or n (%)	p-value	mean (sd) or n (%)	mean (sd) or n (%)	mean (sd) or n (%)	p-value
**Behavioural factors**								
Smoking status								
Non-smoker	185 (88.1%)	121 (83.5%)	161 (86.1%)	0.700	154 (91.1%)	208 (89.7%)	173 (90.6%)	0.364
Yes, occasionally	13 (6.2%)	10 (6.9%)	13 (7.0%)		11 (6.5%)	11 (4.7%)	7 (3.7%)	
Alcohol use								
Low AUDIT-C	152 (72.4%)	89 (61.4%)	125 (66.8%)	0.091	132 (77.7%)	167 (72.0%)	138 (72.3%)	0.382
High AUDIT-C	58 (27.6%)	56 (38.6%)	62 (33.2%)		38 (22.4%)	65 (28.0%)	53 (27.8%)	
**Chronic medical illness**								
HIV positive	99 (47.1%)	69 (47.6%)	91 (48.7%)	0.954	65 (38.2%)	88 (37.9%)	79 (41.4%)	0.742
Hypertensive	29 (13.8%)	21 (14.5%)	16 (8.6%)	0.171	12 (7.1%)	18 (7.8%)	19 (10%)	0.572
Diabetic	10 (4.8%)	3 (2.1%)	7 (3.7%)	0.416	4 (2.4%)	5 (2.2%)	9 (4.7%)	0.259
**Adiposity measures**								
Body mass index	27.9 (6.8)	25.4 (5.4)	24.6 (4.6)	**<0.001**	27.8 (6.9)	28.4 (7.0)	28.4 (7.5)	0.655
Waist circumference	86.3 (14.4)	81.3 (11.3)	78.9 (9.4)	**<0.001**	86.1 (14.6)	87.7 (15.7)	87.5 (16.2)	0.576
Waist hip ratio	0.8 (0.1)	0.8 (0.1)	0.8 (0.1)	**<0.001**	0.8 (0.1)	0.8 (0.1)	0.8 (0.1)	0.770
**Previous trauma**								
Lifetime trauma score	2.0 (1.8)	1.9 (1.8)	1.8 (1.7)	0.354¥	1.0 (1.5)	1.4 (1.7)	1.0 (1.3)	**0.017**¥
Lifetime trauma score: median (iqr)	2.0 (2.0)	1.0 (2.0)	1.0 (3.0)		0.5 (2.0)	1.0 (2.0)	1.0 (2.0)	
Childhood trauma score	16.5 (3.6)	16.2 (3.7)	15.8 (2.7)	0.089¥	15.6 (2.6)	16.1 (2.8)	15.8 (2.3)	0.055¥
Childhood trauma score: median (iqr)	15.0 (3.0)	15.0 (3.0)	15.0 (2.0)		14.0 (2.0)	15.0 (3.0)	15.0 (3.0)	
**Other mental health factors**								
Depression score (CESD)	33.6 (12.2)	33.5 (12.2)	32.0 (13.3)	0.424	12.9 (8.5)	13.6 (9.3)	14.4 (9.9)	0.319
Depression categories								
score: <16	19 (9.1%)	8 (5.5%)	27 (14.4%)	**0.023**	124 (72.9%)	160 (69.0%)	132 (69.1%)	0.642
score:≥16	191 (91.0%)	137 (94.5%)	160 (85.6%)		46 (27.1%)	72 (31.0%)	59 (30.9%)	
**Protective factors**								
Social support score (MSPSS)	34.9 (5.7)	34.5 (4.4)	35.6 (4.4)	0.155	35.2 (4.3)	35.2 (4.4)	34.5 (4.6)	0.197
Perceived stress score (PSS)	23.3 (5.6)	22.3 (5.9)	23.1 (5.7)	0.248	21.5 (4.7)	21.9 (5.1)	21.5 (4.7)	0.567
Resilience score (CD-RISC)	75.4 (6.8)	74.0 (6.0)	73.9 (5.3)	**0.023**	74.9 (5.5)	75.7 (6.2)	75.4 (6.6)	0.410
Rape stigma score (RSS)	20.7 (7.2)	20.3 (6.9)	20.8 (7.1)	0.812				

Footnote: ¥ Kruskal-Wallis test used to assess differences in the adiponectin tertile groups. Values in bold indicate p-value ≤ 0.05. Data are expressed as mean (SD) for continuous variables with normal distribution, median (interquartile range) for continuous variables with skewed distribution, and N(percentage) for categorical variables. Abbreviations: Adiponectin (ADP); Childhood Trauma Questionnaire Short Form (CTQ-SF); Life Events Checklist (LEC); The Connor-Davidson Resilience Scale (CD-RISC); Multidimensional Scale of Perceived Social Support (MSPSS); Perceived Stress Scale (PSS), Rape Stigma Scale (RSS); Alcohol Use Disorders Identification Test- Consumption (AUDIT-C); Center for Epidemiologic Studies Depression Scale (CES-D).

### 3.5 Regression analysis

In multivariate logistic regression (MLR), at the 6-month follow-up, higher s-ADP levels were significantly associated with a reduced risk of probable PTSD in the whole cohort. Specifically, those participants in the highest s-ADP tertile group had a 56% reduced risk of PTSD (aOR = 0.44 (0.22–0.90), p = 0.024) compared to participants in the lowest tertile group (Table 4). Participants in the mid-level s-ADP tertile group had a non-significant 36% reduced risk of probable PTSD as compared to those in the lowest tertile group, (aOR = 0.64 (0.35–1.19), p = 0.158), (Table 4). However, the interaction of adiponectin x rape-exposure was not significant.

**Table 4:** Multivariate logistic regression for probable PTSD at 3- and 6-month follow-up in the whole sample.

	Probable PTSD at 3-months			Probable PTSD at 6-months		
	aOR(95% CI)	p-value	Overall effect of variable/interaction	aOR(95% CI)	p-value	Overall effect of variable/interaction
**Rape Exposure**						
Yes	2.33 (1.61–3.36)	**≤0.001**		0.78 (0.37–1.65)	0.516	
**Adiponectin serum level**			0.685			
2.23–10.56	Ref			Ref		0.065
10.56–14.66	0.89 (0.46–1.74)	0.721		0.64 (0.35–1.19)	0.158	
14.68–32.12	1.10 (0.57–2.21)	0.383		0.44 (0.22–0.90)	**0.024**	
**Adiponectin serum level & exposure group**			0.433			
2.23–10.56 x rape exposure	Ref			Ref		0.255
10.56–14.66 x rape exposure	1.08 (0.67–1.73)	0.763		1.19 (0.47–3.04)	0.708	
14.68–32.12 x rape exposure	1.36 (0.84–2.19)	0.209		2.20 (0.22–0.90)	0.108	
**Baseline facto**rs						
Body mass index	0.99 (0.98–1.01)	0.317		0.96 (0.83–1.12)	0.636	
Lifetime trauma score (LEC)	1.14 (1.08–1.22)	≤0.001		–	–	
Depression Score (CESD)	1.05 (1.04–1.06)	≤0.001		1.04 (1.02–1.06)	**≤0.001**	
Perceived stress score (PSS)	–			0.99 (0.95–1.03)	0.565	
**3-month factors**						
Body mass index	–			1.04 (0.89–1.22)	0.590	
Depression Score (CESD)	–			1.02 (1.00–1.04)	**0.075**	
Perceived stress score (PSS)	–			1.12 (1.06–1.17)	**<0.001**	

Footnote: False discovery rate controlling procedure performed for multiple testing at 10% false discovery rate. The adjusted p-value is 0.03 and significant p-values indicated in bold. Probable PTSD based on DTS ≥40. Abbreviations: Childhood Trauma Questionnaire Short Form (CTQ-SF); Life Events Checklist (LEC); Center for Epidemiologic Studies Depression Scale (CES-D); Perceived Stress Scale (PSS); RE, rape exposure. Baseline factors, as measured at baseline visit; 3-month factors, as measured at 3-month visit.

## 4 Generalized estimating equation (GEE) model

Lastly, we fitted a GEE model with time, baseline s-ADP levels, rape-exposure, and their individual two-way interactions. We adjusted for the time-varying variables of BMI, lifetime trauma score, depression score and perceived stress score (Table 5). Rape exposure was associated with a significant increased risk of probable PTSD (aOR = 10.67 [5.93–19.19], p ≤ 0.001). The mid-level s-ADP tertile category was associated with a significantly reduced risk of probable PTSD at the 6-month follow-up as compared to the lowest s-ADP tertile group (aOR = 0.45 [0.22–0.94], p = 0.035). However, the interaction effect of adiponectin tertile category x RE group was not significant.

**Table 5:** Generalized Estimating Equation model for probable PTSD in the whole sample (N = 1134).

	Adjusted OR (95%CI)	p-value	Overall effect
Time			0.044
Month 0	Ref		
Month 3	1.73 (0.95–3.17)	0.074	
Month 6	2.28 (1.21–4.30)	**0.011**	
**Adiponectin serum level**			0.408
serum levels:2.23–10.56	Ref		
serum levels:10.59–14.66	1.49 (0.83–2.68)	0.180	
serum levels:14.68–32.12	1.25 (0.66–2.37)	0.496	
**Rape Exposure**			
Yes	10.67 (5.93–19.19)	**<0.001**	
**Interaction between time and adiponectin**			0.272
Month 3 & serum levels:10.59–14.66	0.69 (0.35–1.34)	0.270	
Month 3 & serum levels:14.68–32.12	0.77 (0.38–1.56)	0.473	
Month 6 & serum levels:10.59–14.66	0.45 (0.22–0.94)	**0.035**	
Month 6 & serum levels:14.68–32.12	0.49 (0.23–1.05)	0.066	
**Interaction between time and rape exposure**			**0.002**
Month 3 & rape exposed	0.44 (0.24–0.81)	**0.008**	
Month 6 & rape exposed	0.30 (0.16–0.57)	**<0.001**	
**Interaction between adiponectin and rape exposure**			0.659
serum levels:2.23–10.56 & rape exposed	Ref		
serum levels:10.59–14.66 & rape exposed	0.91 (0.49–1.69)	0.770	
serum levels:14.68–32.12 & rape exposed	1.22 (0.65–2.29)	0.543	
**Time varying factors**			
Body Mass Index (kg/m^2^)	1.00 (0.98–1.02)	0.808	
Lifetime trauma score (LEC)	1.59 (1.46–1.73)	**<0.001**	
Depression score (CES-D)	1.10 (1.08–1.11)	**<0.001**	
Perceived stress score (PSS)	1.08 (1.05–1.11)	**<0.001**	

Abbreviations: Childhood Trauma Questionnaire Short Form (CTQ-SF); Life Events Checklist (LEC); Center for Epidemiologic Studies Depression Scale (CES-D); Perceived Stress Scale (PSS). Footnote: Probable PTSD based on a DTS on total score of ≥40. Significant p-values ≤ 0.05 indicated in bold.

### 4.1 Sensitivity analysis

We conducted a sensitivity analysis of the non-imputed and imputed datasets. This yielded comparable findings. Both the mid- and highest s-ADP tertile categories were associated with a significant reduced risk of probable PTSD (aOR = 0.31 [0.21–0.81], p = 0.016; aOR = 0.36 [ 0.14–0.91], p = 0.030). The sensitivity analysis is presented in the Supplementary Table 2 (S2).

### 4.2 Post-hoc analyses

Finally, we examined for possible associations between s-ADP and the three DSM-IV PTSD symptom clusters (Supplementary table, S3). Among the RUE, at 6-month follow-up, a trend towards significance was shown for the mid- and highest-level s-ADP tertile groups and a reduced risk of avoidance symptoms clusters (p = 0.051, p = 0.033). There were no significant associations between s-ADP and any of the three PTSD symptom clusters in the RE group.

## 5 Discussion

In this cohort of black South African women, we found an inverse correlation between s-ADP and prospective risk of probable PTSD in the sample overall. This association persisted even after controlling for traditional PTSD risk factors such as age, social support, childhood, lifetime trauma exposures, as well as adiposity measures. Only a single other study has examined the association between adiponectin and PTSD in humans(Na et al., 2017). The latter study described a statistically significant inverse correlation between plasma adiponectin levels and PTSD severity in a sample of male firefighters. However, key differences between the latter study and the current one should be noted. First, in terms of study population (Japanese vs. Africans) and study design (cross-sectional vs. longitudinal) which confounds direct comparison. Ethnic and gender variations in adiponectin levels have been found in various populations (Mente et al., 2010; Meshkini et al., 2018; Ohman-Hanson et al., 2016). Specifically, lower adiponectin levels in men compared to women have been observed in general population samples (Obata et al., 2013). Second, PTSD was assessed with a different instrument, and plasma, not serum, adiponectin was examined in that study. Circulating adiponectin levels have been shown to differ among individuals with mood and anxiety disorders, dependent on the type of biospecimen (plasma versus serum) examined (Vuong et al., 2020). Despite these differences, the previous study, like ours, demonstrated an inverse association between adiponectin and PTSD.

Several potential mechanisms could explain the higher risk of PTSD associated with lower adiponectin concentrations. Adiponectin receptors (AdipoR1 and AdipoR2) are abundantly expressed in discrete brain regions generally affected by trauma-related disorders, including the amygdala, hypothalamus, cortex, and hippocampus (Thundyil et al., 2012). Adiponectin signalling regulates cognition and synaptic function in the hippocampus, an important area for learning and memory processes (Bloemer et al., 2019). In animal stress models, Zhang et al. (2016) showed that adiponectin-infusions exerted neurotrophic effects in adult rat dentate gyrus (DG) cells of the hippocampus (Zhang et al., 2016). The same research group also found that PTSD mouse models deficient in adiponectin exhibited impaired fear-extinction learning during a classical Pavlovian fear-conditioning paradigm (Zhang et al., 2016). Human studies of PTSD have consistently reported increased concentrations of inflammatory cytokines and imbalances in immune cell production (Cavalcante Passos et al., 2015). Adiponectin suppresses the production of TNF-alpha, a potent proinflammatory cytokine which typically activates the HPA axis (Dunn, 2000) and robust evidence supports a dysfunctional HPA axis in PTSD (Dunlop and Wong, 2019; Speer et al., 2019).

The lack of a significant interaction effect between s-ADP and PTSD among rape exposed participants could be explained by cohort characteristics (e.g metabolic) or other unexamined factors. Adiponectin has been shown to be inversely associated with obesity phenotypes as well as several obesity-related diseases (Achari and Jain, 2017; Nigro et al., 2014). In this study, at baseline, adiposity indices (BMI, WC and WHR) were found to be significantly inversely related to adiponectin levels among the RE, but not the RUE group. There was also a higher prevalence of HIV infection and hypertension among the RE versus RUE participants. Adipokine secretion has been shown to be altered by HIV-infection, HIV-associated lipodystrophy, and antiretroviral therapy (Palios et al., 2012; Sankalé et al., 2006; Tsiodras and Mantzoros, 2006). As cardiometabolic diseases are prevalent among individuals with PTSD, further investigation of metabolic factors within this cohort are warranted. Lastly, results may have been impacted by the timing of adiponectin assays following the rape. Adiponectin levels have been shown to be reduced in the immediate days following other traumas (e.g motor-vehicle accidents, burns) (Pervanidou et al., 2008; Venkatesh et al., 2009), which is hypothesised to relate to adiponectin's interrelationship with serum-cortisol (Fernández-Real et al., 2003). In this sample a significantly higher number of RE participants had baseline adiponectin levels in the lowest adiponectin tertile as compared to the RUE participants.

Although study findings underscore the importance of identifying mental health problems among women who present to health services in the early stages following a sexual assault; high rates of probable PTSD were also observed among RUE participants. This may be explained by study participants being drawn from a population with high levels of chronic trauma exposure. This group of women may have experienced other forms of violence (other than rape), such as physical or emotional intimate partner violence that were not accounted for. In summary, s-ADP levels were inversely associated with probable PTSD over time. Thus, s-ADP testing may allow for early identification of those at risk for PTSD. These individuals may benefit from early psychological interventions that have been shown to be effective in attenuating development of PTSD (Roberts et al., 2019). Given that circulating adiponectin levels are easily measured and may be useful in the early diagnosis of PTSD, its clinical value as a risk biomarker for PTSD and trauma-related disorders remains highly promising.

### 5.1 Strengths and limitations

Strengths of the present study include the prospective design, and a relatively large and homogenoussample, which may reduce confounding. Some limitations were unavoidable and merit consideration. The sample for analysis was drawn from a larger parent cohort study. Although recruited participants were similar on certain socio-demographic factors such as education and food security, RE and RUE groups included in this set of analyses did demonstrate significant differences on other baseline factors, including HIV-status, hypertension, and BMI. These differences may have influenced baseline s-ADP levels. Another consideration is that adiponectin assays were conducted on non-fasting blood samples, though serum adiponectin levels have been shown to not be directly regulated by acute fasting (Gavrila et al., 2003; Merl et al., 2005). A further limitation is that we analysed a single time-point measurement of serum adiponectin levels taken in the days following rape exposure. s-ADP levels may change in the weeks following trauma exposure and the lack of repeated s-ADP assays means than any fluctuation was not captured in these analyses. At baseline, a PTSD diagnosis was established using the MINI, a structured clinical diagnostic interview. At follow-up, PTSD symptoms were determined using the DTS, which is a self-report measure of PTSD. Having a clinical assessment by a psychiatrist or psychologist was not feasible in this limited resource setting and future research should assess clinical evaluation. However, the use of questionnaires is feasible in rape crisis centres and individuals who report many symptoms on these self-report measures can subsequently be referred to an appropriate mental health care provider. Information on the type of chronic medication, dietary factors and levels of physical activity were not collected as part of this study. Serum insulin and fasting glucose levels were not collected as part of this study, conversely, Hb1Ac was used as an alternative measure of glucose tolerance. A high attrition rate in the RE group and those lost to follow-up may have experienced an improvement or deterioration in mental well-being. Lastly, the results of this study cannot be generalized to other ethnic groups or to male participants. It will be necessary to replicate study findings in other large, well characterised longitudinal samples.

## 6 Conclusions

Higher serum adiponectin levels were associated with a reduced risk of probable PTSD, even after adjusting for BMI and other adiposity measures. These results suggest that adiponectin may play a role in the development of PTSD, independent of adiposity. Whether adiponectin is a putative biomarker of risk for PTSD following rape or for PTSD following other traumas, has yet to be established. Future studies are needed to replicate these novel findings, including examination for associations with trauma other than rape and cumulative trauma exposure over time.

## Funding statement

This research is supported by: (i) The South African Research Chair in PTSD (SARChi UID64811) hosted by Stellenbosch University, funded by the 10.13039/501100001342Department of Science and Technology (DST) and administered by the South African National Research Foundation (NRF) and (ii) South African Medical Research Council in terms of the SAMRC's Flagships Awards Project SAMRC-RFA-IFSP-01-2013/RAPE COHORT.

## CRediT authorship contribution statement

**Eileen Vuong:** Conceptualization, Methodology, Data curation, Writing – original draft, Writing – review & editing. **Shibe Mhlongo:** Project administration, Formal analysis, Writing – review & editing. **Esnat Chirwa:** Data curation, Investigation, Formal analysis, Writing – review & editing, Project administration, Writing – review & editing. **Carl Lombard:** Formal analysis, Supervision, Writing – review & editing. **Nasheeta Peer:** Writing – review & editing, Supervision. **Sian Megan Hemmings:** Writing – review & editing, Supervision. **Naeemah Abrahams:** Investigation, Resources, Project administration, Writing – review & editing, Supervision, Funding acquisition. **Soraya Seedat:** Conceptualization, Methodology, Writing – review & editing, Supervision.

## Declaration of competing interest

The authors declare that the research was conducted in the absence of any commercial or financial relationships that could be construed as a potential conflict of interest.

## Data Availability

Data will be made available on request.
