# Supplementary material for: Serum adiponectin-levels are predictive of probable posttraumatic stress disorder in women
Source: Neurobiol Stress. 2022 Aug 8;20:100477. doi: 10.1016/j.ynstr.2022.100477 (PMC9379978; doi:10.1016/j.ynstr.2022.100477)
Supplement: Multimedia component 1 [file mmc1.docx]

**Supplementary Tables**

**S1: Baseline demographic and clinical differences of study participants by retention and follow-up status** **(N=1135)**

|  | **Rape exposed (N=542)** | | | | **Rape unexposed (N=593)** | | | |
| --- | --- | --- | --- | --- | --- | --- | --- | --- |
|  | **Missed 3m or 6m visit** | **Missed 3m & 6m study visits** | **Completed all 3 visits** |  | **Missed 3m or 6m visit** | **Missed 3m & 6m study visits** | **Completed all 3 visits** |  |
|  | n=117 (21.6%) | n=190 (35.1%) | n=235 (43.4%) |  | n=68 (11.5%) | n=46 (7.8%) | n=479 (80.8%) |  |
|  | mean (sd) or n (%) | mean (sd) or n (%) | mean (sd) or n (%) | p value | mean (sd) or n (%) | mean (sd) or n (%) | mean (sd) or n (%) | p value |
| **Age(years)** | 24.7 (5.3) | 24.0 (4.9) | 25.2 (5.5) | 0.075 | 24.6 (5.0) | 25.8 (5.4) | 25.8 (5.5) | 0.229 |
| **Relationship status** |  |  |  |  |  |  |  |  |
| Single | 24 (20.5%) | 31 (16.3%) | 52 (22.1%) | 0.603 | 7 (10.5%) | 8 (17.4%) | 70 (14.6%) | 0.708 |
| Relationship, not cohabiting | 84 (71.8%) | 144 (75.8%) | 162 (68.9%) |  | 56 (83.6%) | 35 (76.1%) | 367 (76.6%) |  |
| Married or cohabiting | 9 (7.7%) | 15 (7.9%) | 21 (8.9%) |  | 4 (6.0%) | 3 (6.5%) | 42 (8.8%) |  |
| **Currently employed** | 28 (23.9%) | 50 (26.3%) | 66 (28.1%) | 0.705 | 9 (13.2%) | 8 (17.4%) | 71 (14.8%) | 0.829 |
| **Completed Secondary school education** | 65 (55.6%) | 112 (59.0%) | 137 (58.3%) | 0.833 | 44 (64.7%) | 31 (67.4%) | 288 (60.1%) | 0.515 |
| **Behavioural factors** |  |  |  |  |  |  |  |  |
| Non-smoker n (%) | 95 (81.2%) | 164 (86.3%) | 208 (88.5%) | **0.035** | 63 (94.0%) | 43 (93.5%) | 429 (89.6%) | 0.337 |
| Yes, occasional smoker | 15 (12.8%) | 12 (6.3%) | 9 (3.8%) |  | 1 (1.5%) | 3 (6.5%) | 25 (5.2%) |  |
| Yes, daily smoker | 7 (6.0%) | 14 (7.4%) | 18 (7.7%) |  | 3 (4.5%) | 0 (0.0%) | 25 (5.2%) |  |
| Alcohol use (AUDIT -C) |  |  |  |  |  |  |  |  |
| Low audit C | 64 (54.7%) | 133 (70.0%) | 169 (71.9%) | **0.003** | 56 (82.4%) | 36 (78.3%) | 345 (72.0%) | 0.149 |
| High audit C | 53 (45.3%) | 57 (30.0%) | 66 (28.1%) |  | 12 (17.7%) | 10 (21.7%) | 134 (28.0%) |  |
| **Chronic medical illness** |  |  |  |  |  |  |  |  |
| HIV positive | 61 (52.1%) | 90 (47.4%) | 108 (46.0%) | 0.544 | 22 (32.4%) | 25 (54.4%) | 185 (38.6%) | 0.054 |
| Diabetes | 3 (2.6%) | 6 (3.2%) | 11 (4.7%) | 0.544 | 5 (7.4%) | 1 (2.2%) | 12 (2.5%) | 0.087 |
| **Adiposity measures** |  |  |  |  |  |  |  |  |
| Body Mass Index | 25.3 (5.3) | 25.1 (4.6) | 27.3 (6.9) | **<0.001** | 26.6 (6.1) | 27.0 (6.6) | 28.6 (7.3) | 0.055 |
| Waist Circumference | 80.8 (11.2) | 80.3 (10.4) | 84.9 (14.0) | **<0.001** | 84.0 (14.0) | 84.6 (12.4) | 87.9 (15.9) | 0.073 |
| **Mental health factors** |  |  |  |  |  |  |  |  |
| Childhood Trauma score (CTQ-SF) | 15.6 (2.3) | 16.2 (3.8) | 16.5 (3.4) | 0.060 | 15.5 (2.5) | 15.5 (2.7) | 15.9 (2.6) | 0.332 |
| PTSD^a^ | 91 (77.8%) | 148 (77.9%) | 190 (80.9%) | 0.695 | 8 (11.8%) | 9 (19.6%) | 60 (12.5%) | 0.379 |
| Perceived stress score (PSS) | 21.2 (6.0) | 23.5 (5.2) | 23.3 (5.7) | **0.001** | 21.8 (5.2) | 21.2 (4.3) | 21.7 (4.9) | 0.804 |
| **Serum adiponectin level** | 12.9 (5.7) | 12.9 (6.1) | 13.1 (5.8) | 0.899 | 12.4 (4.5) | 11.4 (4.3) | 13.2 (4.2) | **0.014** |

Footnote: ^a^Participants were assigned probable PTSD status based on the on DTS on total score of ≥40. Abbreviations: AUDIT-C, Alcohol Use Disorders Identification Test-Concise; m= month; PTSD, posttraumatic stress disorder; s-ADP, serum adiponectin. Significant p-values ≤ 0.05 indicated in bold.

**S2. Sensitivity analyses (General Estimating Equation) comparing imputed data with non-imputed data for key variables.**

|  | **Adjusted OR (95%CI)** | **p-value** |
| --- | --- | --- |
| **Time** |  |  |
| Month 0 | Ref |  |
| Month 3 | 1.39 (0.65-2.99) | 0.400 |
| Month 6 | 2.47 (1.16-5.29) | **0.020** |
| **Adiponectin serum level** |  |  |
| serum levels:2.23-10.56 | Ref |  |
| serum levels:10.59-14.66 | 1.75 (0.81-3.80) | 0.156 |
| serum levels:14.68-32.12 | 1.33 (0.60-2.96) | 0.486 |
| **Rape Exposure** |  |  |
| Yes | 9.59 (4.45-20.68) | **<0.001** |
| **Interaction between time and adiponectin** |  |  |
| Month 3 & serum levels:10.59-14.66 | 0.68 (0.28-1.66) | 0.396 |
| Month 3 & serum levels:14.68-32.12 | 0.87 (0.35-2.14) | 0.761 |
| Month 6 & serum levels:10.59-14.66 | 0.31 (0.12-0.81) | **0.016** |
| Month 6 & serum levels:14.68-32.12 | 0.36 (0.14-0.91) | **0.030** |
| **Interaction between time and rape exposure** |  |  |
| Month 3 & rape exposed | 0.61 (0.27-1.35) | 0.225 |
| Month 6 & rape exposed | 0.43 (0.19-0.97) | **0.043** |
| **Interaction between adiponectin and rape exposure** |  |  |
| serum levels:2.23-10.56 & rape exposed | Ref |  |
| serum levels:10.59-14.66 & rape exposed | 0.97 (0.45-2.11) | 0.944 |
| serum levels:14.68-32.12 & rape exposed | 1.07 (0.48-2.39) | 0.875 |
| **Time varying factors** |  |  |
| Body Mass Index (kg/m^2^) | 1.00 (0.98-1.02) | 0.939 |
| Lifetime trauma score (LEC) | 1.58 (1.42-1.75) | **<0.001** |
| Depression score (CES-D) | 1.10 (1.08-1.12) | **<0.001** |
| Perceived stress score (PSS) | 1.08 (1.05-1.12) | **<0.001** |

Abbreviations: Childhood Trauma Questionnaire Short Form (CTQ-SF); Life Events Checklist (LEC); Center for Epidemiologic Studies Depression Scale (CES-D); Perceived Stress Scale (PSS). Footnote: Probable PTSD based on a DTS on total score of ≥40. Significant p-values ≤ 0.05 indicated in bold

**S3. Post-hoc analyses examining associations between serum adiponectin and PTSD symptom clusters at 3- and 6-month follow-up**

|  | | | | | | | | | |
| --- | --- | --- | --- | --- | --- | --- | --- | --- | --- |
|  | **3-months** | | | | | | | | |
|  | **Rape Exposed: N=312** | | | | | | | | |
| Adiponectin serum level range | Intrusion | | | Avoidance/Numbing | | | Hyperarousal | | |
|  | mean (95% CI) | IRR (95% CI) | p value | mean (95% CI) | IRR (95% CI) | p value | mean (95% CI) | IRR (95% CI) | p value |
| 2.23-10.56 | 4.55 (3.67; 5.44) | 1.00 |  | 7.30 (6.01; 8.58) | 1.00 |  | 6.44 (5.42; 7.46) | 1.00 |  |
| 10.56-14.66 | 4.91 (3.74; 6.09) | 1.08 (0.72; 1.62) | 0.709 | 7.04 (5.72; 8.36) | 0.96 (0.69; 1.34) | 0.831 | 6.62 (5.47; 7.77) | 1.03 (0.74; 1.42) | 0.870 |
| 14.68-32.2 | 5.50 (4.42; 6.57) | 1.21 (0.84; 1.74) | 0.313 | 8.37 (6.94; 9.80) | 1.15 (0.85; 1.55) | 0.372 | 6.81 (5.6; 7.94) | 1.06 (0.79; 1.42) | 0.708 |
|  |  |  |  |  |  |  |  |  |  |
|  | **Rape Unexposed: N=537** | | | | | | | | |
| Adiponectin serum level range | Intrusion | | | Avoidance/Numbing | | | Hyperarousal | | |
|  | mean (95% CI) | IRR (95% CI) | p value | mean (95% CI) | IRR (95% CI) | p value | mean (95% CI) | IRR (95% CI) | p value |
| 2.23-10.56 | 1.27 (0.83; 1.71) | 1.00 |  | 2.85 (2.20; 3.51) | 1.00 |  | 2.90 (2.5; 3.54) | 1.00 |  |
| 10.56-14.66 | 1.25 (0.85; 1.65) | 0.98 (0.55; 1.76) | 0.950 | 2.77 (2.16; 3.38) | 0.97 (0.66; 1.42) | 0.879 | 3.06 (2.48; 3.63) | 1.06 (0.75; 1.48) | 0.751 |
| 14.68-32.2 | 1.57 (1.10; 2.04) | 1.23 (0.68; 2.24) | 0.489 | 3.46 (2.75; 4.18) | 1.21 (0.82; 1.79) | 0.329 | 3.66 (3.04; 4.28) | 1.26 (0.90; 1.78) | 0.181 |
|  |  |  |  |  |  |  |  |  |  |
|  |  |  |  |  |  |  |  |  |  |
|  | **6-months** | | | | | | | | |
|  | **Rape Exposed: N=275** | | | | | | | | |
| Adiponectin serum level range | Intrusion | | | Avoidance/Numbing | | | Hyperarousal | |  |
|  | mean (95% CI) | IRR (95% CI) | p value | mean (95% CI) | IRR (95% CI) | p value | mean (95% CI) | IRR (95% CI) | p value |
| 2.23-10.56 | 3.44 (2.52; 4.36) | 1.00 |  | 5.71 (4.41; 7.02) | 1.00 |  | 5.21 (4.09; 6.34) | 1.00 |  |
| 10.56-14.66 | 3.01 (2.09; 3.94) | 0.88 (0.52; 1.47) | 0.619 | 4.93 (3.68; 6.18) | 0.86 (0.55; 1.35) | 0.519 | 4.59 (3.47; 5.72) | 0.88 (0.58; 1.34) | 0.555 |
| 14.68-32.2 | 3.70 (2.73; 4.67) | 1.08 (0.68; 1.70) | 0.754 | 6.19 (4.89; 7.48) | 1.08 (0.73; 1.61) | 0.696 | 5.55 (4.45; 6.65) | 1.06 (0.73; 1.55) | 0.742 |
|  |  |  |  |  |  |  |  |  |  |
|  | **Rape Unexposed: N=489** | | | | | | | | |
| Adiponectin serum level range | Intrusion | | | Avoidance/Numbing | | | Hyperarousal | | |
|  | mean (95% CI) | IRR (95% CI) | p value | mean (95% CI) | IRR (95% CI) | p value | mean (95% CI) | IRR (95% CI) | p value |
| 2.23-10.56 | 2.14 (1.49; 2.79) | 1.00 |  | 4.02 (3.09; 4.96) | 1.00 |  | 3.69 (2.90; 4.48) | 1.00 |  |
| 10.56-14.66 | 1.42 (0.94; 1.89) | 0.66 (0.36; 1.20) | 0.175 | 2.66 (2.01; 3.30) | 0.66 (0.44; 1.00) | 0.051 | 2.72 (2.13; 3.31) | 0.74 (0.50; 1.09) | 0.122 |
| 14.68-32.2 | 1.23 (0.80; 1.66) | 0.58 (0.31; 1.07) | 0.082 | 2.51 (1.89; 3.13) | 0.62 (0.41; 0.96) | 0.033 | 2.85 (2.19; 3.50) | 0.77 (0.52; 1.15) | 0.205 |

Footnote: False discovery rate controlling procedure performed for multiple testing at 10% false discovery rate. The adjusted p-value is 0.004.

Abbreviations: IRR, Incidence Rate Ratio. PTSD symptom clusters according to DSM-IV PTSD criteria, as measured with the Davidson Trauma Scale.
